# Tioconazole-Loaded Transethosomal Gel Using Box–Behnken Design for Topical Applications: In Vitro, In Vivo, and Molecular Docking Approaches

**DOI:** 10.3390/gels9090767

**Published:** 2023-09-21

**Authors:** Muhammad Imran Qureshi, Qazi Adnan Jamil, Faisal Usman, Tanveer A. Wani, Mudassir Farooq, Hamid Saeed Shah, Hassan Ahmad, Ruqaiya Khalil, Muhammad Sajjad, Seema Zargar, Safina Kausar

**Affiliations:** 1 Department of Pharmaceutics, Faculty of Pharmacy, The Islamia University of Bahawalpur, Bahawalpur 66000, Pakistan; qureshim1122@gmail.com (M.I.Q.); qazi.adnan@iub.edu.pk (Q.A.J.); 2 Department of Pharmaceutics, Faculty of Pharmacy, Bahauddin Zakariya University, Multan 60800, Pakistan; safinakausar555@gmail.com; 3 Department of Pharmaceutical Chemistry, College of Pharmacy, King Saud University, Riyadh 11451, Saudi Arabia; 4 Department of Manufacturing Pharmacy, Faculty of Pharmacy, Mahidol University, Bangkok 10400, Thailand; mudassir.far@student.mahidol.ac.th; 5 Institute of Pharmaceutical Sciences, University of Veterinary and Animal Sciences, Syed Abdul Qadir Jillani (Out Fall) Road, Lahore 54000, Pakistan; hamid.saeed@uvas.edu.pk; 6 Faculty of Pharmaceutical Sciences, University of Central Punjab, 1-Khayabaan-e-Jinnah Road, Johar Town, Lahore 54000, Pakistan; h.ahmad@ucp.edu.pk; 7 Centro De Investigaciones Biomédicas, University of Vigo (CINBO), 36310 Vigo, Spain; ruqaiyakhalil@gmail.com; 8 Department of Biochemistry, Genetics and Immunology, University of Vigo, 36310 Vigo, Spain; 9 College of Pharmacy, University of Sargodha, Sargodha 40100, Pakistan; sajjadawan121@yahoo.com; 10 Department of Biochemistry, College of Sciences, King Saud University, Riyadh 11451, Saudi Arabia; szargar@ksu.edu.sa

**Keywords:** transethosomes, Lipoid S100, Tioconazole, antifungal activity, transethosomal gel, Box–Behnken design

## Abstract

Tioconazole (TCZ) is a broad-spectrum fungicidal BCS class II drug with reported activity against *Candida albicans*, *dermatophytes*, and certain *Staphylococci* bacteria. We report the use of TCZ-loaded transethosomes (TEs) to overcome the skin’s barrier function. TCZ-loaded TEs were fabricated by using a cold method with slight modification. Box–Behnken composite design was utilized to investigate the effect of independent variables. The fabricated TEs were assessed with various physicochemical characterizations. The optimized formulation of TCZ-loaded TEs was incorporated into gel and evaluated for pH, conductivity, drug content, spreadability, rheology, in vitro permeation, ex vivo permeation, and in vitro and in vivo antifungal activity. The fabricated TCZ-loaded TEs had a % EE of 60.56 to 86.13, with particle sizes ranging from 219.1 to 757.1 nm. The SEM images showed spherically shaped vesicles. The % drug permeation was between 77.01 and 92.03. The kinetic analysis of all release profiles followed Higuchi’s diffusion model. The FTIR, DSC, and XRD analysis showed no significant chemical interactions between the drug and excipients. A significantly higher antifungal activity was observed for TCZ-loaded transethosomal gel in comparison to the control. The in vivo antifungal study on albino rats indicated that TCZ-loaded transethosomal gel showed a comparable therapeutic effect in comparison to the market brand Canesten^®^. Molecular docking demonstrated that the TCZ in the TE composition was surrounded by hydrophobic excipients with increased overall hydrophobicity and better permeation. Therefore, TCZ in the form of transethosomal gel can serve as an effective drug delivery system, having the ability to penetrate the skin and overcome the stratum corneum barrier with improved efficacy.

## 1. Introduction

Fungal disorders are among the most frequent dermatological problems in the world, with a high incidence in underdeveloped as well as developed countries. Fungal conditions substantially influence people’s lives, sometimes resulting in significant morbidity and mortality. More than 150 million people are affected by fungal infections. Fungal infections can affect different body parts and are classified accordingly. Mycoses can be classified into superficial, cutaneous, subcutaneous, and systemic. However, superficial mycosis of the skin is one of the most prevalent infectious diseases in clinical settings [1]. It is believed that superficial mycoses affect 20–25% of the population. Superficial fungal infections affect skin structures such as the epidermis, dermis, and inner layer. This creates a need to deliver the drug with the desired therapeutic concentration to a target site such as the epidermis, dermis, or mucosa. For superficial fungal infections, topical therapy is often recommended over systemic treatment because it delivers a therapeutic agent directly to the target site, resulting in fewer adverse effects [2].

Nonetheless, the outermost layer of skin, the stratum corneum (Sc), is the principal barrier to drug permeation. There is a need to develop a drug delivery system that overcomes the stratum corneum’s barriers. Many antifungal creams, gels, and sprays are available for topical treatment. Although these agents are highly effective, many adverse effects are associated with using these formulations, such as stinging, redness, and edema [3]. Furthermore, because of the quick release of the therapeutic agents from these formulations, they might engage the body’s immune system, causing a variety of allergic responses. Deep-seated fungal infections such as aspergillosis and candidiasis are more challenging to treat with standard topical formulations due to the non-availability of the drug at the target site. The inability of antifungal agents to penetrate the various layers of the skin has resulted in strenuous efforts to solve this problem [4].

A wide range of antifungal agents, such as clotrimazole, econazole, miconazole, ketoconazole, and Nystatin, have been employed successfully for inhibiting fungal growth [5]. Tioconazole (TCZ) is a therapeutic agent classified as a BCS class II drug, and it is recognized as a fungicidal agent having a broad spectrum of action against several microorganisms, including *Candida albicans*, *dermatophytes*, and certain *Staphylococci* bacteria. TCZ is approved for topical application to treat superficial mycoses and vaginal candidiasis. TCZ has been utilized at high dosages due to its poor absorption through the skin, resulting in various undesirable effects, such as itching, local irritation, and vaginal burning. TCZ faces a significant challenge in the risk of contact allergy. It is essential to create a cream/gel to minimize such consequences [6].

Many initiatives have been undertaken to enhance the bioavailability of therapeutic agents having low dissolution and permeability rates. Nanoparticle carriers are one of the examples of these initiatives. Because of their ability to increase solubility, bioavailability, and antifungal efficacy, nanoparticles are particularly promising for overcoming these limitations. Furthermore, incorporating therapeutic agents into nanoparticles has the potential to lower their toxicity. Due to their inherent benefits and flexibility over traditional formulations, nanocarrier-based transdermal formulations, for instance, nanocrystals, nanoemulsions, and solid lipid nanoparticles (SLNs), have gained importance for transdermal administration. Liposome carriers, on the other hand, have been explored for topical drug delivery since the 1980s and have provoked the curiosity of researchers. Despite this, typical liposomes do not penetrate deeply into the Sc. As a result, new lipid-based vesicles have been produced as better variants of liposomes throughout the last decade [7].

Transethosomes (TEs) are the latest-generation, ultra-deformable vesicular drug delivery system reported in 2012 by Song et al. [8]. The TE composition includes the ethosome composition (ethanol, phospholipid, cholesterol) and an additional compound such as an edge activator and permeation enhancer. These novel vesicles were fabricated to consolidate the benefits of standard ethosomes and transfersomes in a single formula to fabricate TEs. Many investigations have found that TEs offer advantages over regular ethosomes. As a result, these unique liposomes, which may combine the benefits of transfersomes and ethosomes, would be helpful as an elastic carrier for delivering therapeutic agents to the dermis through Sc barriers [9].

Therefore, in this study, we describe the development and characterization of TCZ-loaded TEs as a potential nanovesicle carrier for topical delivery and loaded into gel formulation to improve TCZ skin permeation. As a result of this innovative technique, TCZ can penetrate deeper into the skin because it bypasses the Sc barrier, directly targeting the fungus in the epidermis and dermis layers, thus minimizing local adverse effects like contact dermatitis.

## 2. Results and Discussion

### 2.1. Box–Behnken Design

**Figure 1:**
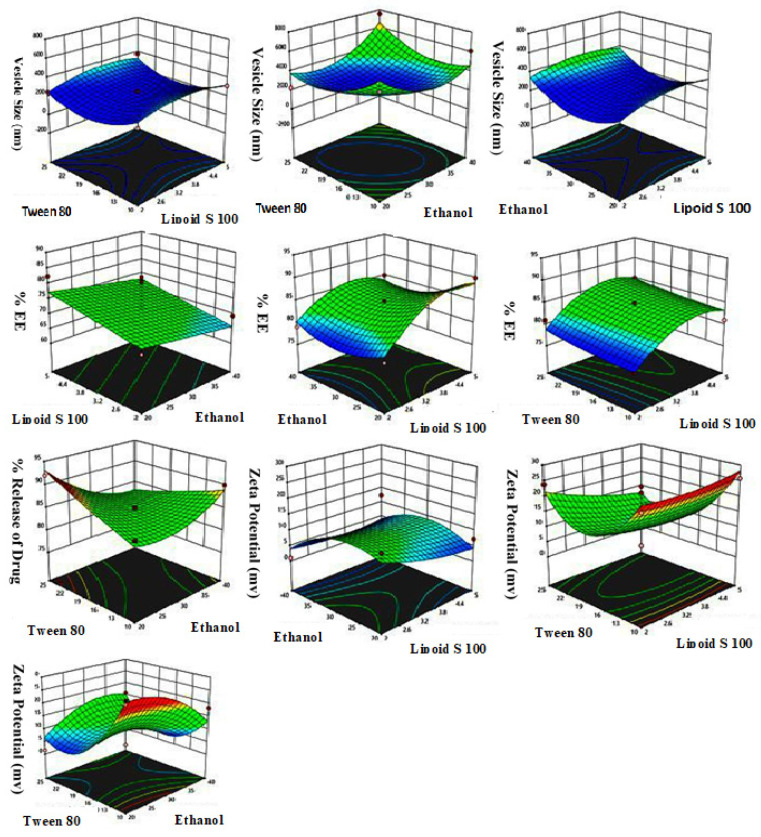
Three-dimensional response surface plots displaying effects of independent variables on vesicle size, % EE, % drug release, zeta potential, and zeta potential.

The Box–Behnken design was utilized to obtain 15 experiment runs with three central points as shown in Table 1. Fabricated TEs were optimized by using design expert software (13.0.5). The dependent variables were vesicle size (Y_1_), % EE (Y_2_), % drug release (Y_3_), and zeta potential (Y_4_). The quadratic model demonstrated the highest degree of fit for the observed data of vesicle size (Y_1_), % drug release (Y_3_), and zeta potential (Y_4_), while the linear model was the most fitted model for the response % EE (Y_2_) of all 15 formulations. The numerical values of R, R^2^, adjusted R^2^, predicted R^2^, standard deviation (SD), and % coefficient of variation (% CV) of each of the four responses are shown in Table 2. The three-dimensional graph in Figure 1 presents the effect of independent variables on vesicle size, % EE drug release, and zeta potential. Moreover, Table 1 computably compares the resultant experimental variables of the responses with the predicted values.

#### 2.1.1. Effects of Independent Variables on Vesicle Size (Y_1_)

The particle sizes of all 15 TCZ-loaded TE formulations were between 219.1 and 757.1 nm. The average particle size was 488.1 nm. The quadratic model is shown in Equation (1).

(1) $$\begin{array}{c} \mathrm{Vesicle}\;\mathrm{size}\;\left(Y_{1}\right)=230.87+28.37X_{1}+30.92X_{2}-8.65X_{3}-10.23X_{1}X_{2}\;+\\ 12.73X_{1}X_{3}+110.03X_{2}X_{3}-70.57X_{1}^{2}+165.18X_{2}^{2}+133.88X_{3}^{2} \end{array}$$

As per the polynomial equation, Lipoid S100 and ethanol positively affected vesicle size. At the same time, Tween 80 negatively affected the particle size. The vesicle size of TE07 was 235.3 nm and increased to 757.1 nm in TE08 when the concentration of Lipoid S100 was increased from 2 to 3.5% *w*/*v*. Similar responses were observed in formulations TE13 and TE07 with vesicle sizes of 248.5 nm and 235.3 nm, respectively. Increased vesicle sizes from 432.1 nm and 378.5 nm in formulations TE05 and TE14 were observed due to a rise in phospholipid concentration from 2 to 5% *w*/*v*. The particle size of TEs was increased with the concentration of phospholipids because the high concentration of phospholipids gives more space in the core of the TEs; hence, more of the drug will be loaded [10,11]. Upon an increase in the concentration of Lipoid S100, the particle size of TEs was increased. This effect was also due to the formulation of multi-lamellar vesicles, previously reported by Harbi et al. and Ahmed et al. [12,13]. The particle size was decreased upon an increase in the concentration of Tween 80. This effect may be due to the reduced surface tension, which results in phospholipids’ arrangement in nanovesicles [14].

Upon an increase in ethanol concentration from 20% to 30% *v*/*v*, it was observed that the particle size of TE vesicles was reduced due to the drop in TE membrane thickness because of the interpenetration of ethanol with the hydrocarbon chain in the vesicular lipid bilayers [15,16]. However, at a high ethanol concentration of 40% *v*/*v* or above, the TE vesicle size increased, and TEs became leaky. Higher ethanol concentrations lowered the vesicle membrane’s thickness [17].

#### 2.1.2. Effects of Independent Variables on % Entrapment Efficiency (Y_2_)

In all 15 TE formulations, the % EE lies between 60.56 and 86.13%, with a mean value of 73.34%.

(2) $${\%\;\mathrm{EE}\;Y}_{2}=71.47+3.57X_{1}-2.11X_{2}+7.30X_{3}$$

The linear model Equation (2) indicates that the phospholipids and surfactant positively affected the % EE. By contrast, ethanol negatively affected the % EE of TCZ-loaded TE formulations. An increase in % EE was observed with increasing Lipoid S100 concentration from 2 to 3.5% in TE04. Similar responses were observed in TE15, TE13, TE06, and TE07, which had 2% *w*/*v* Lipoid S100 and showed % EE of 69.3, 74.87, 68.24, and 63.34% respectively. The % EE increased to 82.34% and 80.71% in formulations TE05 and TE01 with an increase in Lipoid S100 to 5% *w*/*v*. Similar findings were also reported in Sildenafil-loaded transferosomes by Ahmed et al. [18]. The initial increase in % EE was due to the rise in phospholipid concentration because of the lipophilic nature of TCZ, as lipophilic drugs have more affinity towards the lipophilic core of TEs [19].

Ethanol showed an inverse effect on the % EE of TCZ-loaded TE formulations. At a low concentration of ethanol, 20% *v*/*v* in formulations TE04 and TE05, the % EE was found to be 86.13 and 82.34%. Conversely, with the rise in ethanol concentration to 40% *v*/*v* in formulations TE12, TE08, and TE03, the % EE decreased to 73.43%, 70.35%, and 67.34%. At a high ethanol concentration, the TEs become leaky; as a result, they showed a lower % EE [20]. Tween 80 was shown in direct relation with % EE. With the lower concentration of Tween 80 in TE2, TE03, TE07, and TE09, the % EE was found to be 65.12, 67.34, 63.34, and 60.56%. The % EE was increased to 86.13, 70.35, and 83.39% in TE04, TE08, and TE14 with the increase in the concentration of Tween 80 from 10% *w*/*w* to 25% *w*/*w* of total phospholipid contents.

#### 2.1.3. Effects of Independent Variables on % Drug Release (Y_3_)

The % drug release lies between 77.01% and 92.03%, with a mean value of 84.85% in all TE formulations. Equation (3) shows the relationship of % drug release.

(3) $$\begin{array}{c} \%\;\mathrm{Drug}\;\mathrm{release}\;\left(Y_{3}\right)=+84.33+3.12X_{1}-1.25X_{2}+0.875X_{3}-1.25X_{1}{\;X}_{2}\;+\\ 0.00X_{1}{\;X}_{3}-3.75X_{2}{\;X}_{3}-3.67X_{1}^{2}+2.58X_{2}^{2}+0.33X_{3}^{2} \end{array}$$

The phospholipids and surfactant showed a positive effect on % drug release, while ethanol showed the inverse effect according to the polynomial equation. A rise in % drug release was observed with an increase in Lipoid S100 from 2 to 3.5%, as shown in TE01, TE03, TE04, TE06, TE07, and TE15. However, the drug release was decreased after a further increase in Lipoid S100 concentration from 3.5 to 5%, as shown in Table 1. As the concentration of phospholipids increased, the rigidity of the vesicle increased as reported by Gonzalez-Rodriguez et al., 2005, resulting in a decrease in the permeation of drugs from the vesicles [21]. The % release was enhanced with an increase in ethanol concentration. The higher fluidity that occurs in the bilayer membrane of TEs could potentially be attributed to the elevated concentration of ethanol [20].

#### 2.1.4. Effects of Independent Variables on Zeta Potential (Y4)

In all TE formulations, the zeta potential lies between −1.04 mV and −28.5 mV, with a mean value of 14.77 mV as evaluated by Equation (4).

(4) $$\begin{array}{c} \mathrm{Zeta}\;\mathrm{potential}\;\left(Y_{4}\right)=+9.59-1.75X_{1}-1.85X_{2}-5.11X_{3}+1.73X_{1}X_{2}\;-\\ 2.63X_{1}X_{3}+6.15X_{2}X_{3}+1.22X_{12}-4.96X_{2}^{2}+11.37X_{3}^{2} \end{array}$$

According to the polynomial equation, the phospholipids, ethanol, and surfactant showed an inverse effect on zeta potential. Negative zeta potential was due to the high ethanol concentration in TE vesicles. Ogiso et al. 2001 previously reported that negatively charged vesicles showed improved skin permeation compared to positively charged vesicles [21].

### 2.2. FTIR Studies

**Figure 2:**
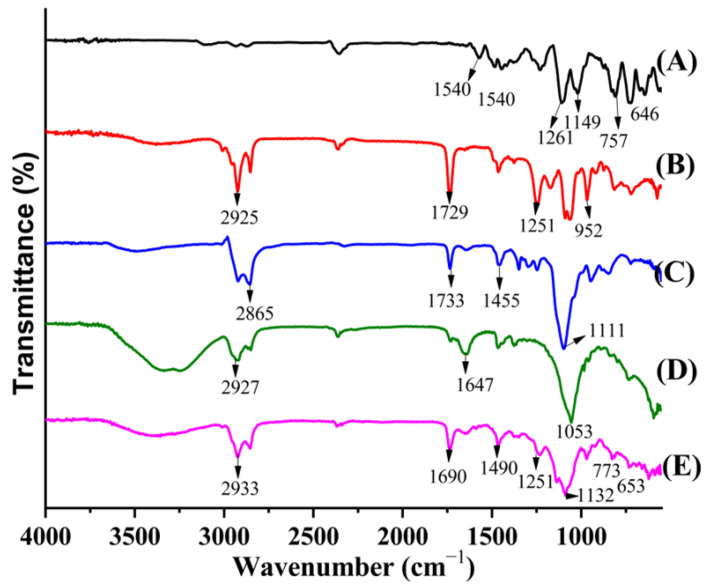
(**A**) Drug, (**B**) Lipoid S100, (**C**) Tween 80, (**D**) cholesterol, (**E**) freeze-dried TE04.

The TCZ FTIR spectrum was similar to the reference of the standard TCZ (KBr discs) of BP 2010 (British Pharmacopeia Commission, 2010). The characteristic peaks corresponding to ν(C=N) of the imidazole group at 1540 cm^−1^, δ(C–H) at 1484 cm^−1^, ν(C=C) at 1428 cm^−1^, ν(C–N) at 1261 cm^−1^, ν(C–O–C) at 1149 cm^−1^, ν(C–S) at 757 cm^−1^, and ν(C–Cl) at 646 cm^−1^ were identified [22,23]. For Lipoid S100, the characteristic band at 1729 cm^−1^ of (C=O) stretching vibration was attributed to the ester group (Figure 2, spectrum A). The presence of C–H bonds in the phospholipids, which were observed at 2925 cm^−1^, and several sharp peaks from 1251 cm^−1^ to 952 cm^−1^ correspond to the phosphate group region (Figure 2, spectrum B) [24].

The O–H stretching vibration of the terminal –OH group of Tween 80 was at 3455 cm^−1^, indicating intermolecular hydrogen bonding among the Tween 80 molecules. The band at 1111 cm^−1^ was attributable to the ethylene oxide unit’s C–O–C stretching vibration in Tween 80. The C–H stretching vibration of the ethylene oxide units in Tween 80 appears at 2865 cm^−1^ (Figure 2, spectrum C). The significant bands identified for the cholesterol molecule were found at 3400 cm^−1^, 2927 cm^−1^, 2895 cm^−1^, 2865 cm^−1^, 1465 cm^−1^, 1440 cm^−1^, 1376 cm^−1^, 1647 cm^−1^, 1055 cm^−1^, 1021 cm^−1^, 984 cm^−1^, 842 cm^−1^, and 800 cm^−1^. The bands between 2800 and 3000 cm^−1^ are characterized as being due to asymmetric and symmetric stretching vibrations of CH_2_ and CH_3_ groups. Cholesterol has one double band (C=C) in the second ring obviously shown at 1647 cm^−1^ (Figure 2, spectrum D). The peak at 1647 cm^−1^ for the double bond in the second cholesterol ring agrees with the results of Zheng et al. [25]. The sharp peak at 1055 cm^−1^ can be attributable to the deformation of control rings.

The spectra of TE04 showed characteristic peaks corresponding to known peaks of *ν*(C=N) of the imidazole group of TCZ at 1690 cm^−1^, δ(C–H) at 1490 cm^−1^, *ν*(C=C), *ν*(C–N) at 1251 cm^−1^, *ν*(C–O–C) at 1132 cm^−1^, *ν*(C–S) at 773 cm^−1^, and *ν*(C–Cl) at 653 cm^−1^. The spectra showed no significant interaction between the drug and excipients observed in formulation TE04. The spectra of TE04 showed a slight shift in intensity in the characteristic peaks of the drug, Lipoid S100, and Tween 80. Overall, characteristic peaks of the drug, Lipoid S100, and Tween 80 were found in the spectra of TE04, indicating not much interaction occurs, as shown in Figure 2, spectrum E. 

### 2.3. Transmission Electron Microscopy (TEM)

**Figure 3:**
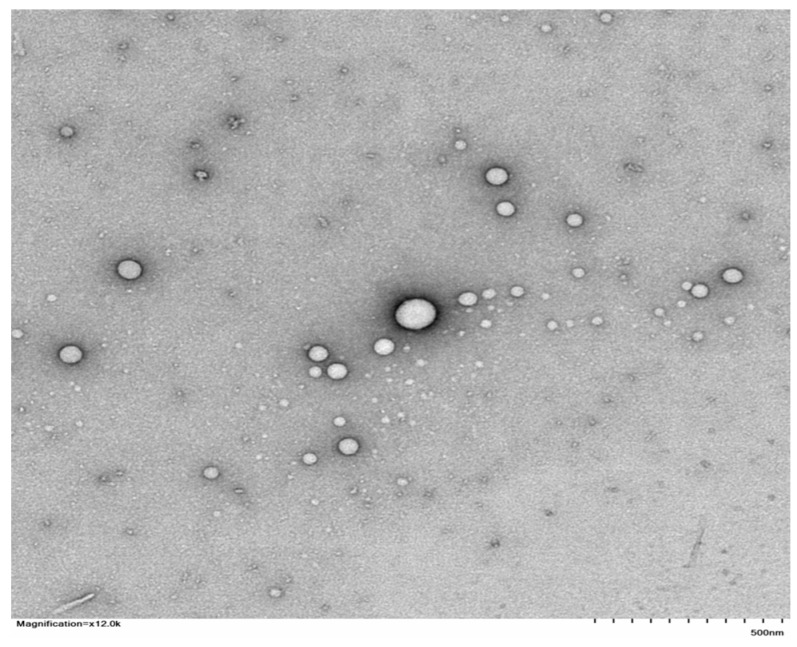
TEM image of TCZ-loaded TE04 formulation; magnification = ×12.0 k (500 nm).

TEM (Hitachi 7700, 120 kv, Ibaraki, Japan) was used to evaluate the shape of the optimized TCZ-loaded TE04 formulation in its initial suspension phase. Figure 3 shows TEM images of the TCZ-loaded TE04 formulation, which was spherical. The vesicles shown in the TEM image were smaller than those noticed using the dynamic light-scattering approach with the Malvern Zetasizer (Malvern Instruments, Enigma Business Park UK). This finding might be due to the solvent-removal effect during specimen preparation for TEM imaging, which can cause size variations. This description was previously reported by Das and Chaudhury et al., who demonstrated that sample preparation might directly influence particle size and structure [26]. A similar observation was made during the size and morphological analysis of finasteride microspheres [27].

### 2.4. X-ray Diffraction (XRD) Analysis

**Figure 4:**
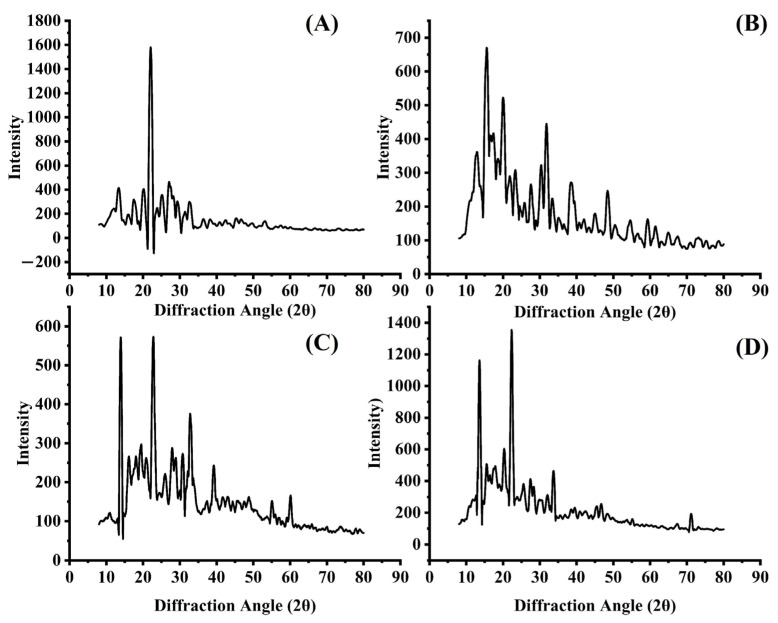
XRD diffraction patterns of (**A**) TCZ, (**B**) cholesterol, (**C**) physical mixture of excipients, and (**D**) freeze-dried TE04.

X-ray diffraction analysis was performed to assess the crystallinity of the resulting formulations, and the profiles are presented in Figure 4. The pure TCZ had a diffraction intensity of 4083.36 at 22.34°, indicating that the substance is crystalline [28]. Diffraction peaks at 2Ɵ were also recorded at 8.61°, 10.39°, 13.79°, 13.27°, 13.93°, 16.35°, 17.15°, 17.23°, 18.31°, 20.43°, 20.49°, 24.51°, 24.41°, 25.53°, 25.67°, 26.37°, 27.41°, 27.63°, 28.79°, 28.19°, 29.39°, 29.23°, and 32.51°. TCZ nanovesicles, after subsequent dehydration, resulted in the conversion of the crystalline form of TCZ into an amorphous form, confirmed by the diminution of the crystalline peaks. These findings followed those confirmed by differential scanning calorimetry (DSC), validating the theory that the TCZ inside the TEs was in an amorphous state [29].

### 2.5. DSC Studies

**Figure 5:**
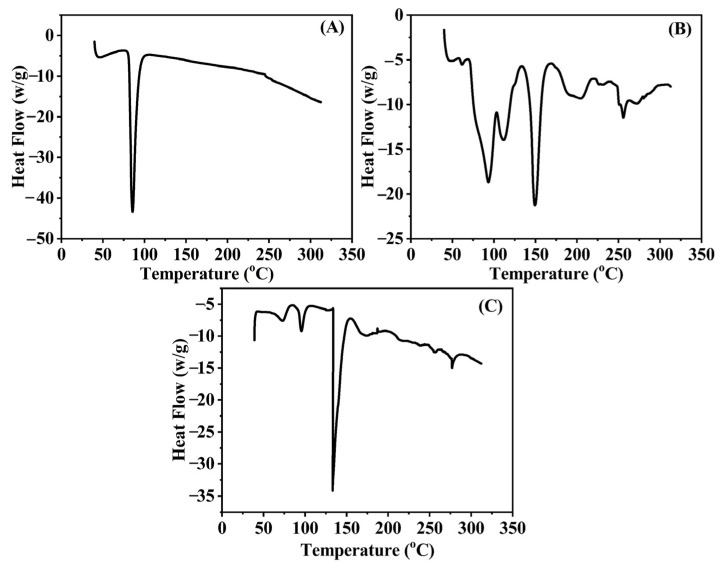
DSC curves of TCZ (**A**), cholesterol (**B**), and freeze-dried TE04 (**C**).

DSC of TCZ displayed a sole endothermic peak at 83.8 °C, with an onset temperature of 77.6 °C associated with melting [28]. This result agrees with the value stated at 82 °C in the literature (British Pharmacopeia Commission, 2013). Two sharp endothermic peaks were detected in the Lipoid S100 DSC analysis. The initial peak manifested at a temperature of 94 °C, whereas the subsequent peak emerged at a temperature of 150 °C, as shown in Figure 5. The observed peaks are indicative of the phase transitions or melting points of distinct constituents present in Lipoid S100. In freeze-dried TE04, the shift in the sharp endothermic peak of the drug to a small peak from 82 to 72 °C indicates the drug was converted from a crystalline to an amorphous state. However, in the case of phospholipids, the sharp melting peak that appeared at 150 °C and 94 °C and shifted to 133 °C and 96 °C might correspond to phase transitions or physical state changes in TCZ and Lipoid S100 in the formulation.

### 2.6. Characterization of Fabricated TCZ-Loaded Transethosomal Gels

#### 2.6.1. Physical Appearance, pH, and Conductivity

The TCZ-loaded transethosomal gels (TEG1–TEG15) were visually consistent in texture, smooth, and milky. The pH of TEGs was 6.73–6.82, within the typical pH range of skin, preventing skin irritation upon application [30,31]. TEG04 showed the most negligible pH value, 6.73. Meanwhile, TEG13 has an almost neutral pH. The conductivity values were in the range of 912–975 µs/cm. Results are shown in Table 3.

#### 2.6.2. Drug Content, Spreadability, and Extrudability

TEG01, TEG04, TEG06 and TEG23 showed a drug content of 98–99%. The spreadability of TEG01, TEG04, TEG06, and TEG23 was 5.28 to 5.75 g/s, while the extrudability was found to be 8.35 to 8.64 g/cm^2^. TEG06 showed the lowest spreadability, and TEG04 showed high spreadability. The results for spreadability and extrudability are shown in Table 3.

#### 2.6.3. Rheological Studies

**Figure 6:**
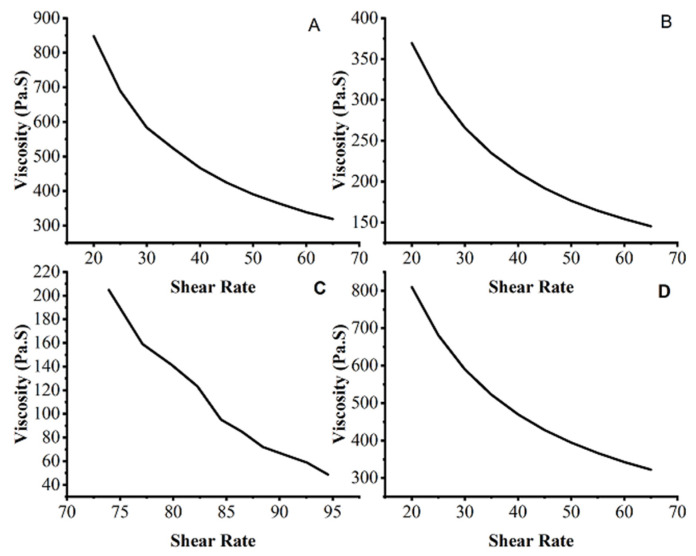
Rheograms of transethosomal gel formulations TEG01 (**A**), TEG04 (**B**), TEG06 (**C**), and TEG13 (**D**).

The rheological investigations of the manufactured TEGs were carried out because viscosity affects the materials’ flow behavior throughout the mixing, packing into containers, storage stability, and transdermal application. The rheograms showed that viscosity dropped as the shear rate increased, as shown in Figure 6 [32]. It was observed that the relationship between viscosity and shear rate is inverse. The TEG formulations demonstrated non-linear viscosity and shear rate behavior, indicating non-Newtonian behavior.

In the current study, the TEG is a shear thinning system, with a decrease in viscosity when shear stress is applied, improved skin spreadability, and ease of application. When the viscosity of fresh and previously developed formulations was contrasted with that of formulations stored at different storage temperatures at various time intervals, a slight decrease was observed, showing that the prepared formulations were stable [33].

### 2.7. In Vitro Drug Release

**Figure 7:**
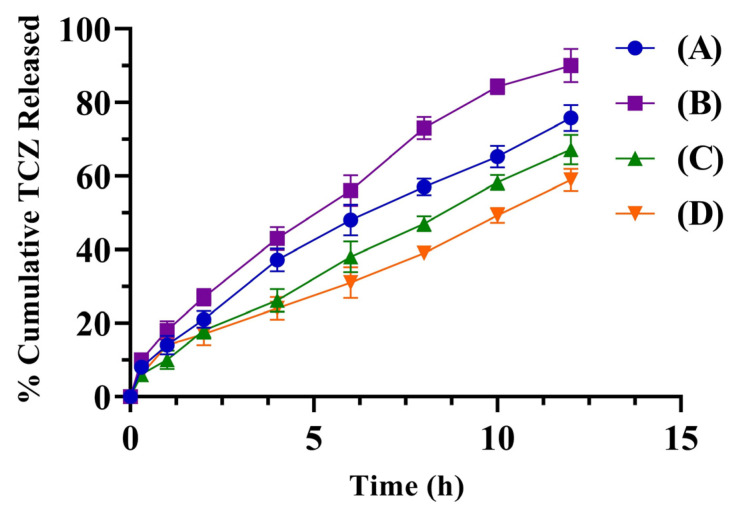
The % cumulative TCZ of TEG01 (A), TEG04 (B), TEG06 (C), and TEG13 (D) that permeated through the cellophane membrane in 12 h.

In vitro drug release revealed that TEG01 and TEG04 containing 3.5% *w*/*v* Lipoid S100, penetrated the skin more than the TEG06 and TEG13 formulations. The TEG01 and TEG04 formulations were chosen as the optimized formulations, with a release of up to 75.81 and 90.05%, respectively, after 12 h. The in vitro % released of TCZ was calculated. TEs were discovered to be a more adequate formulation for deeper layer permeation than other deformable vesicles. Figure 7 depicts the percentage of in vitro cumulative TCZ that penetrated through the cellophane membrane in 12 h for TEG01, TEG04, TEG06, and TEG13 samples.

### 2.8. Ex Vivo Permeation Studies

**Figure 8:**
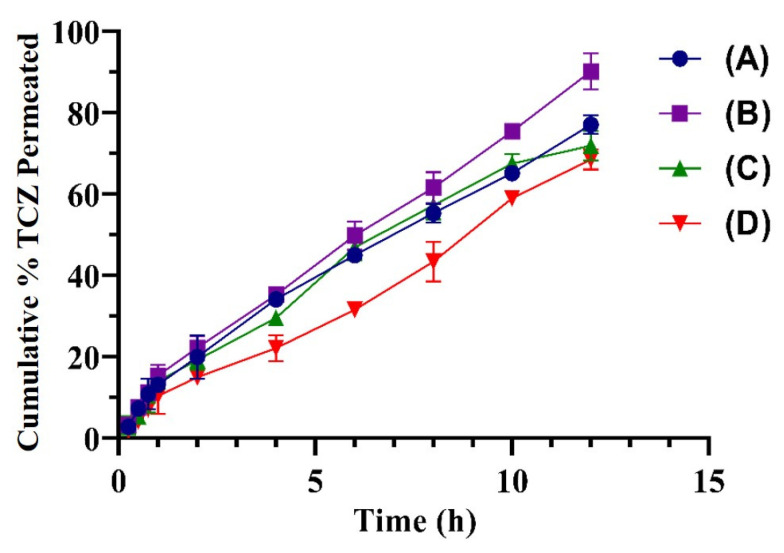
The % cumulative TCZ of TEG01 (A), TEG04 (B), TEG06 (C), and TEG13 (D) that permeated through albino rat skin in 12 h.

TCZ deposition and permeation were examined ex vivo on rabbit skin using vertical Franz diffusion cells at 37 °C. TEG04 showed a maximum drug permeation of 92.12% after 12 h. On the other hand, the TEG13 formulation showed a drug release of up to 66.19%, as shown in Figure 8. The J_max_ (permeation flux) and enhancement ratio (ER) of pure TCZ gel and the TCZ-loaded TEG04 gel formulation are shown in Table 4.

### 2.9. In Vitro Drug Release Kinetics

The drug release kinetics followed the Higuchi model, which is characterized by a Fickian diffusion process. The observed phenomenon could be attributed to the enhanced fluidity of the bilayer membrane of TE, which is likely a result of the elevated concentration of ethanol [34]. A similar result was observed in the development of pentoxifylline transfersomes [27].

### 2.10. In Vitro Antifungal Activity

The antifungal activity effects against *Aspergillus fumigatus* and *Candida albicans* are presented in Table 5. A substantial decline was found in the MIC and MFC of TCZ-loaded TE04 and TEG04 compared to those of the ethanolic solution; the TCZ TE04 formulation showed superior antifungal activity compared to the ethanolic solution of the pure drug and the TCZ-loaded TEG04 formulation. Blank samples showed no antifungal activity. The previously stated MIC and MFC of TCZ for *Candida albicans* and *Aspergillus fumigatus* were 3.1 µg/mL and 5.7 µg/mL, respectively [35].

### 2.11. Stability Study

A slight change in drug content was observed in both TE and TEG formulations. However, particle size was increased to some extent. This may be due to aggregation over time. The pH was in an acceptable range for topical application and remained homogeneous after storage for 6 months, as presented in Table 6 and Table 7.

### 2.12. In Vivo Antifungal Activity

**Figure 9:**
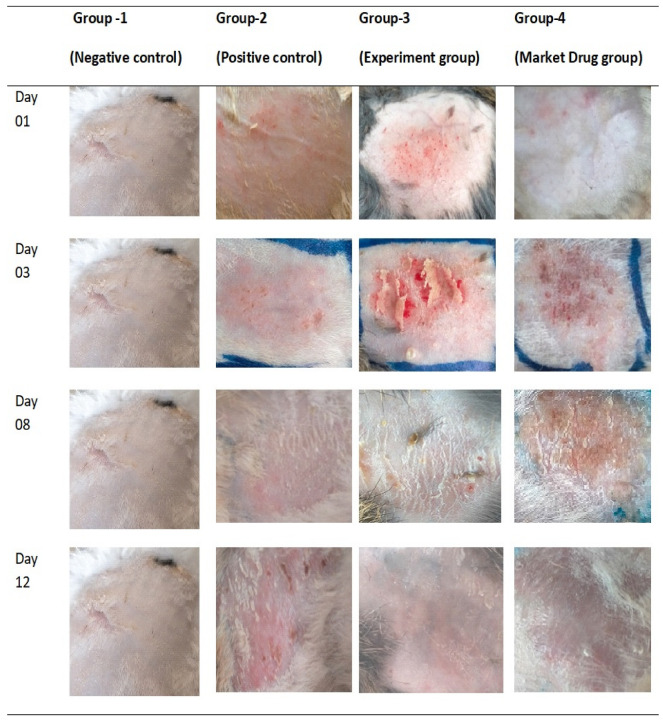
Photographs of different sections of rabbit skin before and after induction of *Candida albicans* on day 1, day 3, day 8, and completion of therapy on day 12.

All albino rats selected for the experiment had typical skin structures, with no clinical signs or symptoms of fungal infection, like inflammation, edema, cracking, or color changes. When the albino rats were infected with a fungus, they developed purple or grey lesions, edema, inflammation, and cracking or scaling of the skin. TEG04 demonstrated considerable in vitro antifungal activity. The standard marketed cream Canesten^®^ was utilized as an external control, while the blank gel was employed as an internal control. Bleeding was observed in the blank-gel-treated albino rats after 8 days, whereas healing of lesions and wounds was observed on the 4th day in groups treated with TCZ-loaded TEG04 or commercialized antifungal cream. Edema and inflammation subsided following therapy with Canesten^®^ cream 1%, but the scales persisted, as shown in Figure 9. At the same time, the rabbit skin that received TCZ-loaded TEG04 showed normal skin with no inflammation. Albino rats treated with TCZ-loaded TEG04 or commercialized antifungal cream recovered completely from infection on day 12; however, albino rats treated with blank gel did not recover by day 12.

### 2.13. Histopathological Examination

**Figure 10:**
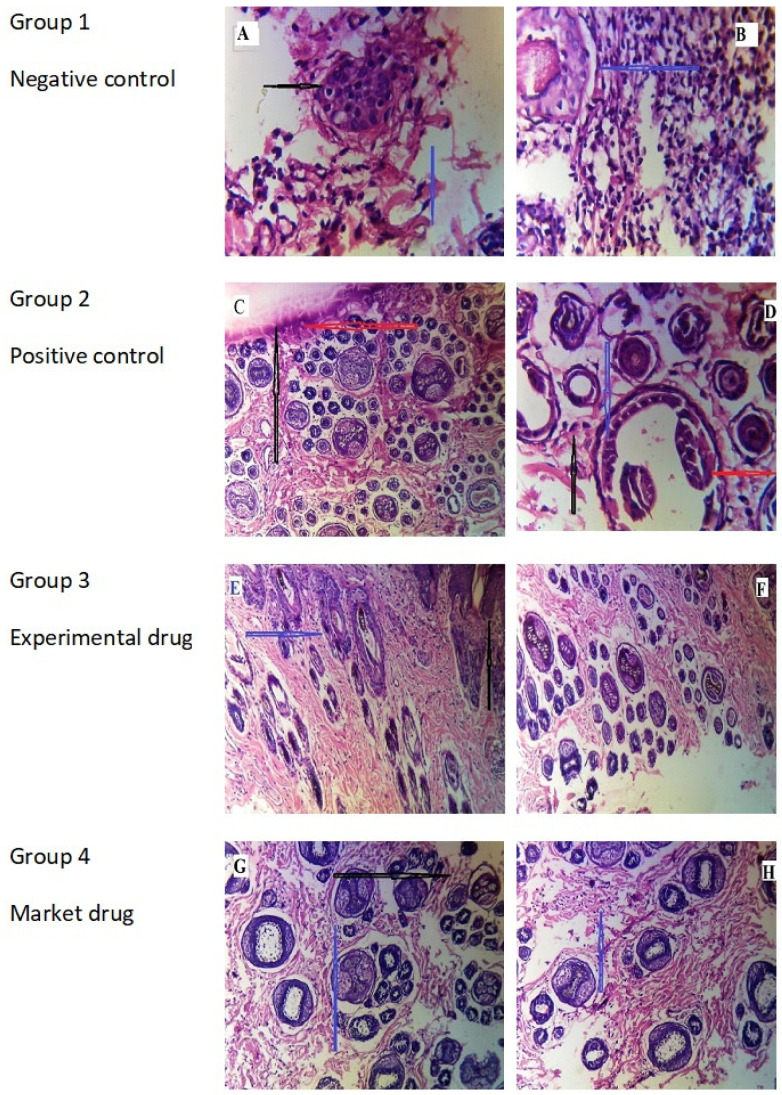
Histopathological variations in skin fungal infection of albino rats using *Candida albicans*. Histological images ((**H**) and (**E**) ×20 and 40) for the negative control (Group 1, (**A**) and (**B**)), positive control (Group 2 (**C**) and (**D**)), Canesten^®^ (Group 3 (**E**) and (**F**)), and TCZ-loaded TEG04 (Group 4 (**G**) and (**H**)) cream 1%.

The negative control group, normal albino rats, showed a uniform dermis and epidermis (black and blue arrows, A) without any structural changes in rabbit skin, as shown in the figure. In contrast, the positive control group exhibited focal acanthosis (C) characterized by mild to severe hyperkeratosis (D), as illustrated by the red and black arrows, respectively. In contrast, the presence of fungal hyphae was observed on the outermost layer of the epidermis, shown by a blue arrow (D). Focal interface dermatitis was additionally found and denoted by a red arrow (D). The dermis layer of rabbit skin was dense, which is a sign of chronic inflammation (black arrow, C), as shown in the figure. The Group 4 albino rats that received Canesten^®^ cream 1% showed better improvement in the dermis and epidermal layer, as shown in the figure. Conversely, the presence of localized acanthosis (shown by the blue arrow, G) and skin appendages (indicated by the black arrow, G) were noted. The Group 3 albino rats that received TCZ-loaded TEG04 gel 1% showed uniform structure in both the epidermis and the dermal layer, as shown in Figure 10. The results seen in Group 3 rabbit skin may be due to the flexible nature of TEs allowing the TCZ to permeate through the different layers of the skin, such as the epidermis and dermis, as compared to Canesten^®^ cream 1% having free drug with a low capability to penetrate through the skin [36]. The results, as mentioned above, were also supported by a quantitative analysis of pharmacological actions using the scoring system as reported by Mona Qushawy et al. and Satyam SM et al. [37,38] and are shown in Table 8.

### 2.14. Molecular Modeling

**Figure 11:**
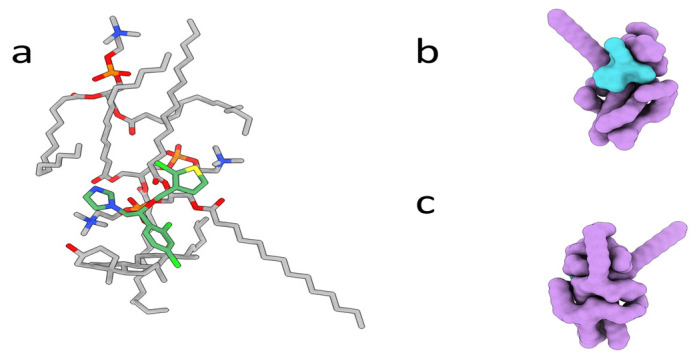
The molecular architecture of TE04 complex. Panel (**a**) presents the excipients as gray sticks, carbon sticks, and TCZ as green sticks. Panels (**b**,**c**) show the molecular surface of the complex. The excipients are shown as purple, and TCZ is shown as a blue surface.

The molecular architecture of the TCZ formulations was established using in silico molecular modeling techniques. This analysis revealed a crucial insight: within the composition of TCZ within TEs, TCZ is enveloped by hydrophobic excipients, as visually represented in Figure 11. This spatial arrangement amplifies the overall hydrophobicity of the formulation, resulting in enhanced membrane permeation. This enhanced permeation, in turn, explains the observed increase in the antifungal activity of the TE formulation. Significantly, our findings align with the FTIR results, which provide evidence of the absence of substantial polar interactions between the excipients and the TCZ molecule. The complex charge complementarity between the thiazole group of TCZ and the polar groups in the excipient may play a role in stabilizing the formulation or influencing its release characteristics. These interactions, along with other hydrogen bonds, facilitate complexation and reduce the overall surface area and gyration radius. As a result, the neutralization of the charge profile of TCZ and the TE formulation enhances permeation across biological membranes, potentially affecting its effectiveness against fungal targets. In an aqueous environment, such as inside the cytoplasm, the exposed chloride moieties can interact with surrounding water molecules, forming hydrogen bonds and causing subsequent solvation. This interaction leads to a decrease in compactness, exposing the drug to potential interactions or reactions.

## 3. Conclusions

Optimized TCZ-loaded TEs were successfully fabricated by utilizing the Box–Behnken composite design. The optimized TEs were spherical, negatively charged, highly flexible nanovesicles with high drug EE and % drug release. The optimized TCZ-loaded TEs were incorporated into gel and evaluated for ex vivo drug release, molecular simulation, physicochemical considerations, in vitro antifungal activity, and in vivo antifungal activity in an animal model. The optimized TCZ-loaded TE formulation showed more significant antifungal activity against *Candida albicans* compared with a TCZ ethanol solution and the TCZ-loaded TEG formulation in albino rats. Finally, it is concluded that fabricated TCZ-loaded TEG can be highly useful in treating superficial mycosis and can provide a potential benefit in treating various superficial fungal infections.

## 4. Materials and Methods

### 4.1. Materials

TCZ was obtained from Biopharm chemical limited, China, and Lipoid S100 (phosphatidylcholine) was obtained from Lipoid GmbH (Ludwigshafen, Germany) as a gift. Tween 80, cholesterol, ethanol, and propylene glycol (PG) were purchased from Sigma-Aldrich (Spruce St., St. Louis, MI, USA).

### 4.2. Fabrication of TCZ-Loaded TEs

TCZ-loaded TEs were prepared using the cold method as reported, with slight modification [10]. Cholesterol, Lipoid S100, Tween 80, and TCZ were dissolved in a specified amount of ethanol. The aqueous phase was added dropwise with continuous stirring at 750 rpm. The TCZ-loaded TE suspension was subjected to sonication to reduce the particle size of the TEs. The TCZ-loaded TE preparations were stored at 4 °C in closed containers for further analysis.

### 4.3. Box–Behnken Composite Design

The Box–Behnken design was selected as an experimental design to formulate the TCZ-loaded TEs and to analyze the effect of excipients on vesicle size, entrapment efficiency, zeta potential, and % of drug release. The Box–Behnken design was used to evaluate the effect of three variables as Equation (5). Table 9 represents the factors and their responses used in the Box–Behnken design.

Y = b_o_ + b_11_X_1_ + b_12_X_2_ + b_13_X_3_ + b_22_X_1_X_2_ + b_23_ X_1_X_3_ + b_24_ X_2_X_3_ + b_32_ X_1_^2^ + b_33_X_2_^2^ + b_34_X_3_^2^ (5)

Y is the measured response to each component level combination; b_0_ is constant; b_11_, b_12_, and b_13_ are linear coefficients; and b_22_, b_23_, and b_24_ are three-variable interaction coefficients. b_32_, b_33_, and b_34_ are quadratic coefficients of observable levels X_1_, X_2_, and X_3_, along with experimental values of independent variables. Independent variables were Lipoid S100 (X_1_), ethanol (X_2_), and Tween 80 (X_3_). Vesicle size (Y_1_), % entrapment efficiency (Y_2_), drug release (Y_3_), and zeta potential (Y_4_) were dependent variables. TCZ-loaded TEs with different constituent compositions are given in Table 10.

### 4.4. Characterization of Fabricated TCZ-Loaded TEs

#### 4.4.1. Particle Size, Zeta Potential, and Polydispersity Index (PDI)

The drug release, physical stability, and cellular absorption of vesicles are all affected by particle size and shape. Light scattering is essential for determining the properties of colloidal and macromolecular dispersions. Wet laser diffraction sizing is used primarily to evaluate the characteristics of TEs and is called dynamic light scattering (DLS) [10].

This approach yields a volume-based primary particle-size distribution. Polydispersity can be measured in terms of uniformity or the degree to which the distribution is symmetric around the median point and the width of the distribution. Small PDI values of 0.1 suggest a homogeneous dispersion, but large values >0.3 imply substantial heterogeneity [39]. The charge on the vesicular dispersion can influence the formulation’s stability and vesicle interaction with the delivery site. It affects long-term physical stability [40]. Laser Doppler anemometry with a Zetasizer Nano-Z instrument (Malvern Instruments, UK) was used to evaluate zeta potential [41].

#### 4.4.2. Fourier Transform Infrared Spectroscopy

An infrared absorption spectrum from Fourier transform infrared spectroscopy (FTIR) identifies chemical interactions in molecules. The spectra create a chemical fingerprint that may identify components. The TCZ–TE interaction was studied using FTIR. An infrared spectrophotometer was used to acquire 20 scans of pure TCZ, phospholipid, surfactant, cholesterol, and lyophilized formulation (TE04) FTIR spectra between 500 cm^−1^ and 4000 cm^−1^.

#### 4.4.3. % Entrapment Efficiency (% EE)

The % EE was assessed quantitatively by ultracentrifugation at high revolution [42]. Briefly, 1 mL of prepared formulations of TCZ-loaded TEs was placed in a centrifuge machine. The samples were centrifuged at 12,000 rpm for 60 min at 4 °C. The supernatant fluid contained the free drug while vesicles settled down in the bottom as a solid layer [43]. The supernatant fluid was separated, filtered with a syringe filter of 0.2 µm, and diluted with ethanol. A UV-Vis spectrophotometer (Perkinelmer lambda 25, Rodgau, Germany) was used to analyze the samples at 220 nm. The % EE of TCZ was calculated using Equation (6).

(6) $$\%\;\mathrm{EE}\;=\frac{\mathrm{Actual}\;\mathrm{amount}\;\mathrm{of}\;\mathrm{TCZ}\;-\;\mathrm{Estimated}\;\mathrm{amount}\;\mathrm{of}\;\mathrm{TCZ}\;\mathrm{in}\;\mathrm{supernatant}\;\mathrm{fluid}}{\mathrm{Actual}\;\mathrm{amount}\;\mathrm{of}\;\mathrm{TCZ}}\times 100$$

#### 4.4.4. In Vitro Drug Release

The drug release study was conducted using a USP dissolution apparatus II (Zhenzhou Lab instrument Equipment Co, Ltd., Zhengzhou Henan, China). The cellophane membrane, having a molecular weight of 12–14,000 Daltons, was steeped for 24 h in distilled water. Then, 1 mL TE samples were loaded in a cellophane bag by tying both ends of the cellophane membrane and treated in the USP dissolution apparatus II in 500 mL of pH 6.4 phosphate-buffered saline (PBS) solution at 100 rpm. At regular time intervals, 5 mL of the sample was taken from the dissolution apparatus and replaced with an equal amount of PBS buffer of pH 6.4. These samples were analyzed for drug release using a UV-Vis spectrophotometer at 220 nm.

#### 4.4.5. Optical Microscopy

The TE sample was dispersed on a glass slide and covered with a cover slip. The specimens were examined, and photographs were taken.

#### 4.4.6. Transmission Electron Microscopy (TEM)

The morphology of the advanced formulation was investigated using transmission electron microscopy (Hitachi 7700, 120 kv, Ibaraki, Japan). A small quantity of the solution was applied onto a grid that had been coated with carbon, and it was left undisturbed for a duration of 5 min to facilitate enhanced adsorption of the nanovesicles onto the carbon layer. The sample was subjected to analysis with the addition of a small quantity of phosphor tungstic acid.

#### 4.4.7. Differential Scanning (DSC)

DSC is a crucial tool for estimating probable interactions between the active ingredients and excipients and the mechanism of transdermal permeation during the pre- and post-formulation characterization of nanoparticles.

The thermal behavior of pure TCZ, cholesterol, and freeze-dried TCZ-loaded TE formulations were evaluated (Shimadzu DSCTA-50 ESI, Tokyo, Japan). Each sample was weighed and placed in an aluminum crucible in a nitrogen atmosphere. The produced samples were heated at 25 to 300 °C.

#### 4.4.8. X-ray Powder Diffraction (XRD)

An XRD investigation of vesicular carrier formulations can be performed to determine whether the carriers are crystalline or amorphous [44,45].

The diffraction patterns of pure TCZ and the freeze-dried TCZ-loaded TE formulation were studied using a D/max 2500 Rigaku powder X-ray diffractometer (Tokyo, Japan) to evaluate drug crystallization behavior. The specimens’ diffraction patterns were obtained at 0.5 °C/min.

#### 4.4.9. Storage Stability of Transethosomes

UV-Vis spectroscopy was used to evaluate the TCZ content in TEs and transethosomal gel for up to 6 months of storage at 2–8 °C. Equation (7) was used to calculate the percentage of TCZ.

(7) $$\begin{array}{c} \%\;\mathrm{TCZ}\;\mathrm{after}\;\mathrm{storage}\;=\left(\mathrm{Concentration}\;\mathrm{of}\;\mathrm{TCZ}\;\mathrm{at}\;\mathrm{time}\;\left(t\right)\right)/\\ (\mathrm{Concentration}\;\mathrm{of}\;\mathrm{TCZ}\;\mathrm{at}\;\mathrm{time}\;(t_{o})\times 100 \end{array}$$

where (TCZ)_t_ and (TCZ)_t_o__ are the TCZ concentration in the formulations measured after 6 months of storage at 2–8 °C and at time t_o_.

### 4.5. Preparation of TE Gel

The TCZ-loaded TE gel formulation (TEG) was fabricated by soaking 0.8% *w*/*v* Carbopol 940 in double-distilled water. The Carbopol 940 was allowed to hydrate for 24 h, and the dispersion was kept at 25 °C. The fabricated TCZ-loaded TE suspension was added to Carbopol 940 (Sigma-Aldrich, Spruce St., St. Louis, MI, USA). Finally, the Carbopol 940 and TE dispersion was stirred at 300 rpm to obtain uniform mixing and a smooth texture. Triethanolamine was added dropwise to neutralize the gel [46].

#### 4.5.1. Physical Appearance, Conductivity, and pH Measurement

The prepared TCZ-loaded TEG was kept in transparent containers to observe the visual appearance, clarity, phase separation, and lump formation. The pH of the gel plays an essential role in the drug’s solubility, stability, and shelf life. The conductivity and pH of the TCZ-loaded TEG were assessed using a PHS-500 pH meter (Hangzhou Lohand Technology, Hangzhou, China) and a DDS-22 conductivity meter (Hangzhou Lohand Technology, Hangzhou, China) at 25 °C.

#### 4.5.2. Extrudability and Spreadability

The ability to spread on the skin surface is one of the critical parameters for gel. A gel formulation must have ideal spreadability. The spreadability of prepared TCZ-loaded TEG was measured by using two glass slides. One slide was fixed while the other glass slide was moved over the selected slide with the application of standard weight. The midpoint of the glasses was marked, and a gel sample was placed at this point [20,47]. Spreadability was calculated using Equation (8).

(8) $$\mathrm{Spreadability}=\frac{\mathrm{Weight}\;\mathrm{of}\;\mathrm{Gel}\;\mathrm{in}\;\mathrm{grams}}{\mathrm{Time}\;\mathrm{taken}\;\mathrm{by}\;\mathrm{gel}\;\left(s\right)}$$

Extrudability is vital in determining the ease of removal and application of products such as ointments, creams, and gels. Extrudability was calculated by extruding a calculated amount of gel from aluminum tubes by applying constant weight to the gel at time t [48].

#### 4.5.3. % Drug Content

The drug content percentage in TEG formulations was determined using a UV-Vis spectrophotometer. A gel formulation containing 1 g of TCZ was diluted with ethanol, and the resulting solution’s absorbance was measured at a wavelength of 220 nm.

#### 4.5.4. Rheological Studies

The rheological studies of TEG formulations were performed using a Brookfield Rheometer (DV-II Ultra, Hadamar-Steinbach, Germany) at 25 °C. The fabricated gels’ viscosity behavior under the application of stress was noted [49].

### 4.6. Ex Vivo Permeation Studies

Ex vivo skin permeability of TEG and the blank gel was assessed on a Franz diffusion cell (PERME GEAR, USA) with a diffusion area of 1.54 cm^2^ on the donor compartment. The ex vivo skin permeation examination was carried out using fresh abdomen skin from albino rats. The skin hair was cleared with a razor blade. Before the next step, the skin was detached from the excised abdominal skin using forceps and cleaned with PBS. The skin was sited between the donor and receptor chambers of the cell, with the Sc layer towards the donor chamber. The receptor chamber was filled with 5 mL of pH 6.5 PBS. In the donor chamber, 1 g of TCZ-loaded TEG, corresponding to 10 mg of TCZ, was inserted. At 37 ± 0.5 °C, the receptor medium was agitated with a magnet [49]. The samples were obtained from the receptor chamber at regular intervals and replaced with an equivalent quantity of fresh PBS, pH 6.5. The concentration of TCZ in the gathered samples was determined using a UV-Vis spectrophotometer at 220 nm. The spectrophotometric analysis involved the calculation of the cumulative amount of TCZ that penetrated through the skin per unit area (g/cm^2^). Equations (9)–(11) were used to compute the other parameters, such as the flux (g/cm^2^/h), permeability coefficient (cm/h), and enhancement ratio:

(9) $$\mathrm{Flux}\;\left(J\right)=\frac{\mathrm{Quantity}\;\mathrm{of}\;\mathrm{TCZ}\;\mathrm{permeated}\;}{\mathrm{time}\;\times \;\mathrm{diffusion}\;\mathrm{area}\;}\times 100$$

(10) $$\mathrm{Permeability}\;\mathrm{coefficient}\;\left(\mathrm{KP}\right)=\frac{\mathrm{Flux}\;\mathrm{of}\;\mathrm{TCZ}=\mathrm{loaded}\;\mathrm{TEG}\;}{\mathrm{Initial}\;\mathrm{amount}\;\mathrm{of}\;\mathrm{TCZ}\;\mathrm{in}\;\mathrm{the}\;\mathrm{donor}\;\mathrm{compartment}}\times 100$$

(11) $$\mathrm{Chamber}\;\mathrm{Enhancement}\;\mathrm{ratio}\;\left(\mathrm{ER}\right)=\frac{\mathrm{Flux}\;\mathrm{of}\;\mathrm{TCZ}-\mathrm{loaded}\;\mathrm{TEG}\;}{\mathrm{Flux}\;\mathrm{of}\;\mathrm{TCZ}\;\mathrm{plain}\;\mathrm{gel}}\times 100$$

### 4.7. Drug Release Kinetics

Drug release behavior from prepared TCZ-loaded TEG was studied by applying different kinetics models for elaborating the mechanism of drug release either by diffusion, erosion, or swelling. The zero-order, first-order, Higuchi, and Korsmeyer–Peppas release models are calculated using Equations (12)–(15).

(12) $$F_{t}=K_{O}t$$

(13) $$l_{n}\left(1-F\right)=-K_{1}t$$

(14) $$F=K_{2}t_{1/2}$$

(15) $$\frac{M_{t}}{M}=K_{3}t_{n}$$

Initial fractions F_t_ and F represent the release of a fraction of the substance at time “t”. The zero, first, Higuchi, and Korsmeyer–Peppas release constants are K_o_, K_1_, K_2_, and K_3_. M, M_t_, and n represent the equilibrium water absorption mass, time (t), and release exponent.

### 4.8. In Vitro Antifungal Activity

The determination of minimal inhibitory concentration (MIC) values for the TCZ-loaded TEG04 formulation, ethanolic solution of TCZ, and TCZ-loaded TEs was conducted using the Micro Broth dilution assay, following the guidelines given by NCCLS (2001) [50]. The experiments were conducted using Sabouraud dextrose broth and Mueller–Hinton broth as the test media. The microplates were inoculated with standard strain suspensions containing 10^6^ colony-forming units per mL. The plates containing the fungus strain were subjected to incubation at a temperature of 30 °C for a duration of 48 h. The inoculum was introduced onto microdilution plates with drug solutions ranging from 0.1 µg/mL to 35 µg/mL. The plates were then placed in an incubator at a temperature of 30 ± 5 °C for a duration of 1 to 2 days. The minimal fungicidal concentration (MFC) is a metric used to quantify the lowest concentration of a medicine required to effectively eliminate fungal organisms.

### 4.9. In Vivo Antifungal Activity

Male albino rats (*n* = 12) were collected from the animal house, faculty of pharmacy, Bahauddin Zakariya University, Multan, and divided into four groups (*n* = 3) to evaluate the in vivo antifungal activity of the optimized TCZ-loaded TEG04 formulation. The albino rats were divided into four groups: negative control (Group 1), positive control (Group 2), experimental (Group 3), and standard (Group 4). Group 1 was not given any drug and received no induction of fungal infection. In Group 2, fungal infection was induced, and a placebo containing blank Carbopol gel was applied. Fungal infections were caused in Groups 3 and 4 using TEG04 and a market formulation (Canesten^®^). Each rabbit was kept in a separate cage with a standard diet.

*Candida albicans* was taken as an infectious agent to infect 9 albino rats of Group 2, Group 3, and Group 4. The fungal species collected were grown on nutrient agar plates and stored in a refrigerator at 4 °C. Each rabbit’s dorsal region was depilated. Fresh cultured contagious inoculum of *Candida albicans* was smoothly rubbed on the depilated skin of albino rats and left for two days, the period necessary to develop the first indications of active infection like redness and scales. The therapy began on day 3, and each formulation was given to the albino rats once a day. The treatment continued until the albino rats wholly recovered from the infection. The antifungal activity of formulation was assessed daily by macroscopic inspection of the affected region.

### 4.10. Molecular Modeling

The molecular architecture of the TE compositions was established with molecular modelers. The molecular structures were sketched using ChemDraw Ultra and later charged and minimized in Molecular Operating Environment (MOE) under the MMFFF94x force field for small molecules. The compounds were docked and later minimized to obtain a stable structure.

## Figures and Tables

**Table 1:** Responses in Box–Behnken design of TCZ-loaded TE formulations (mean ± SD, *n* = 3).

		Independent Variables			Dependent Variables			
Run	Code	X_1_ (% *w*/*v*)	X_2_ (% *v*/*v*)	X_3_ (% *w*/*w*)	Y_1_ (nm)	Y_2_ (%)	Y_3_ (%)	Y_4_ mV
01	TE01	3.5	30	17.5	254.4 ± 2.13	80.71 ± 0.21	85.12 ± 0.54	−21.2 ± 0.23
02	TE02	05	30	10.0	314.4 ± 3.21	65.12 ± 0.84	81.23 ± 0.35	−25.8 ± 0.34
03	TE03	3.5	40	10.0	610.3 ± 1.14	67.34 ± 0.54	90.14 ± 0.45	−18.2 ± 0.56
04	TE04	3.5	20	25.0	229.5 ± 2.19	86.13 ± 0.46	92.03 ± 0.67	−1.50 ± 0.23
05	TE05	05	20	17.5	432.1 ± 3.26	82.34 ± 0.35	90.04 ± 0.41	−7.19 ± 0.56
06	TE06	02	20	17.5	402.7 ± 3.98	68.24 ± 0.45	79.12 ± 0.37	−13.9 ± 0.21
07	TE07	02	30	10.0	235.3 ± 2.24	63.34 ± 0.97	77.01 ± 0.59	−24.3 ± 0.56
08	TE08	3.5	40	25.0	757.1 ± 2.54	70.35 ± 0.54	82.24 ± 0.61	−15.8 ± 0.43
09	TE09	3.5	20	10.0	522.8 ± 3.65	60.56 ± 0.21	85.43 ± 0.54	−28.5 ± 0.23
10	TE10	3.5	30	17.5	219.1 ± 4.21	63.43 ± 0.56	84.02 ± 0.71	−3.79 ± 0.67
11	TE11	3.5	30	17.5	219.1 ± 2.45	63.43 ± 0.24	84.02 ± 0.34	−3.79 ± 0.98
12	TE12	05	40	17.5	227.8 ± 3.43	73.43 ± 0.56	85.21 ± 0.67	−1.27 ± 0.43
13	TE13	02	30	25.0	248.5 ± 1.54	74.87 ± 0.23	81.24 ± 0.23	−23.8 ± 0.78
14	TE14	05	30	25.0	378.5 ± 5.23	83.39 ± 0.54	85.21 ± 0.56	−14.8 ± 0.23
15	TE15	02	40	17.5	239.3 ± 2.45	69.3 ± 0.35	79.29 ± 0.32	−1.04 ± 0.43

**Table 2:** Summary regression analysis for responses Y_1_, Y_2_, Y_3_, and Y_4_.

Fit Model	Response	R^2^	Adjusted R^2^	Predicted R^2^	SD	% CV
Quadratic	Vesicle size (Y_1_)	0.6547	0.0331	−4.4953	162.73	46.13
Linear	% EE (Y_2_)	0.5842	0.4708	−0.3307	6.04	8.47
Quadratic	% Drug release (Y_3_)	0.9198	0.7755	−0.2474	2.05	2.44
Quadratic	Zeta potential (Y_4_)	0.7749	0.3697	−0.5651	7.84	57.38

**Table 3:** pH, conductivity, % drug content, spreadability, and extrudability values of TCZ-loaded TEG01, TEG04, TEG06, and TEG13 formulations (mean ± SD, *n* = 5).

No.	Code	pH	Conductivity (µs/cm)	% Drug Content	Spreadability (g/s)	Extrudability (g/cm^2^)
01	TEG01	6.79 ± 0.03	913± 0.03	98.15 ± 0.21	5.28 ± 0.15	8.35 ± 0.05
02	TEG04	6.73 ± 0.01	974± 0.02	99.24 ± 0.31	5.75 ± 0.15	8.64 ± 0.01
03	TEG06	6.61 ± 0.04	975± 0.03	98.64 ± 0.15	5.22 ± 0.15	8.53 ± 0.05
04	TEG13	6.82± 0.03	912± 0.01	98.86 ± 0.15	5.34 ± 0.15	8.44 ± 0.07

**Table 4:** J_max_ (permeation flux) and enhancement ratio (ER) of TCZ plain gel and TCZ-loaded TEG04 gel formulation.

Formulation Code	J_max_ (µg cm^−2^ h^−1^)	K_p_ (cm h^−1^)	ER
TCZ gel	6.8	0.53	5.2
TEG04	24.1	3.1	

**Table 5:** MIC and MFC of drug and formulations in µg/mL (mean ± SD, *n* = 3).

Fungal Species	Code	MIC (µg/mL)	MFC (µg/mL)
*Aspergillus fumigatus*	TCZ	5.8 ± 0.12	34.2 ± 0.14
	TE04	4.3 ± 0.54	21.6 ± 0.25
	TEG04	5.3 ± 0.98	24.1 ± 0.37
*Candida albicans*	TCZ	2.8 ± 0.23	24.4 ± 0.45
	TE04	1.9 ± 0.34	17.2 ± 0.67
	TEG04	2.3 ± 0.18	19.8 ± 0.21

Tioconazole (TCZ), transethosomal formulation (TE04), and transethosomal gel (TEG04).

**Table 6:** Stability study of transethosomes (TE04) (mean ± SD, *n* = 3).

Time	Drug Content	Particle Size
Initial	99.54 ± 0.14	229.5 ± 2.34
After 2 months	98.53 ± 0.32	234.5 ± 3.12
After 4 months	98.10 ± 0.21	238.1 ± 4.41
After 6 months	98.21 ± 0.15	240.4 ± 3.64

**Table 7:** Stability study of transethosomal gel (TEG04) (mean ± SD, *n* = 3).

Time	Drug Content	pH	Homogeneity
Initial	99.24 ± 0.26	6.8 ± 0.25	Homogeneous
After 2 months	98.53 ± 0.53	6.5 ± 0.04	Homogeneous
After 4 months	98.10 ± 0.64	6.9 ± 0.19	Homogeneous
After 6 months	98.21 ± 0.35	7.1 ± 0.31	Homogeneous

**Table 8:** A scoring system based upon the histopathological evaluation of the antifungal effect of TEG04 compared to the commercial brand (Canesten^®^ 1%).

Group	Fungal Hyphae	Hyperkeratosis	Acanthosis	Interface Dermatitis	Inflammation	Dermis
Group 1 (Negative control)	0	0	0	0	0	0
Group 2 (Positive control)	1	1	1	1	2	1
Group 3 (Experimental drug)	0	0	0	0	0	0
Group 4 (Market drug)	0	0	1	0	0	0

Fungal hyphae: 0 = absent, 1 = present; hyperkeratosis: 0 = absent, 1 = mild, 2 = remarkable. acanthosis: 0 = absent, 1 = focal, 2 = diffuse; interface dermatitis: 0 = absent, 1 = mild, 2 = remarkable; inflammation and dermis: 0 = homogeneous, 1 = mild inflammation, 2 = dense chronic inflammation.

**Table 9:** Dependent and independent variables for TCZ-loaded TEs used in the Box–Behnken design.

Variables	Level	
	Low	High
Independent variables		
X1: Phospholipids (Lipoid S100) % *w*/*v*	2	5
X2: Ethanol % *v*/*v*	20	40
X3: Surfactant (Tween 80) % *w*/*w* of total Lipoid S100	10	25
Dependent Variables		
Y1: Vesicle size (nm)		
Y2: % Entrapment Efficiency		
Y3: % Drug release		
Y4: Zeta potential (mV)		

**Table 10:** Experimental runs for TCZ-loaded TEs with different compositions of ingredients.

Run	Phospholipids % *w*/*v*	Ethanol % *v*/*v*	Surfactant % *w*/*w* of Total Lipids	Cholesterol % *w*/*v*	Drug % *w*/*v*
01	3.5	30	17.5	0.5	1
02	05	30	10.0	0.5	1
03	3.5	40	10.0	0.5	1
04	3.5	20	25.0	0.5	1
05	05	20	17.5	0.5	1
06	02	20	17.5	0.5	1
07	02	30	10.0	0.5	1
08	3.5	40	25.0	0.5	1
09	3.5	20	10.0	0.5	1
10	3.5	30	17.5	0.5	1
11	3.5	30	17.5	0.5	1
12	05	40	17.5	0.5	1
13	02	30	25.0	0.5	1
14	05	30	25.0	0.5	1
15	02	40	17.5	0.5	1

## Data Availability

The data presented in this study are available from the authors upon reasonable request.
